# Hyperspectral optical imaging of two different species of lepidoptera

**DOI:** 10.1186/1556-276X-6-369

**Published:** 2011-05-04

**Authors:** José Manuel Medina, Sérgio Miguel Cardoso Nascimento, Pete Vukusic

**Affiliations:** 1 Centre of Physics, University of Minho, Campus de Gualtar, Braga, 4710-057, Portugal; 2 School of Physics, University of Exeter, Stocker Road, Exeter, EX4 4QL, UK

## Abstract

In this article, we report a hyperspectral optical imaging application for measurement of the reflectance spectra of photonic structures that produce structural colors with high spatial resolution. The measurement of the spectral reflectance function is exemplified in the butterfly wings of two different species of Lepidoptera: the blue iridescence reflected by the nymphalid *Morpho didius *and the green iridescence of the papilionid *Papilio palinurus*. Color coordinates from reflectance spectra were calculated taking into account human spectral sensitivity. For each butterfly wing, the observed color is described by a characteristic color map in the chromaticity diagram and spreads over a limited volume in the color space. The results suggest that variability in the reflectance spectra is correlated with different random arrangements in the spatial distribution of the scales that cover the wing membranes. Hyperspectral optical imaging opens new ways for the non-invasive study and classification of different forms of irregularity in structural colors.

## Introduction

Many insects and birds contain photonic structures self-assembled at the nanometer scale, some of which produce structural colors. Structural colors are the result of the manipulation of the flow of light due to coherent scattering associated with the presence of various forms of photonic crystal [1]. Their reflectance spectra depend markedly on both the illumination and the viewing angle providing special visual effects such as iridescence and the basis to develop novel photonic applications and nanomaterials [1]. In comparison with colored materials composed of absorbing dyes and pigments, structural colors usually contain periodic nanostructures embedded in the irregularity of the microstructure. Irregularity-based structures play a fundamental role in non-iridescent coloration and in surface color appearance [1]. Their study demands new optical methods that can combine spectral variability together with detailed spatial properties. For this purpose, reflectance spectra recorded using spectrophotometer-based systems are not adequate because they may average over large diameter spots [2,3]. Hyperspectral imaging is a common spectroscopy technique in non-invasive sensing analysis. From a stack of images taken at a series of narrow-bandwidth wavelengths, hyperspectral imaging provides a three-dimensional array or "image cube," with two spatial dimensions and the third the spectral axis [4,5]. In comparison with multispectral methods, in hyperspectral imaging the distance between adjacent wavelengths is less than the spectral bandwidth providing the continuous spectral reflectance function pixel by pixel.

The aim of this study was to measure the reflectance spectra of different structurally colored butterfly wings using hyperspectral imaging. Previous studies on multispectral imaging have analyzed the colorimetric properties of synthetic interference coatings [6]. Therefore, it is not clear whether hyperspectral optical imaging is adequate for reflectance estimation and the research on the irregularity of structurally colored systems. A specific aim of this investigation was the conversion of the estimated reflectance spectra of butterfly wings into perceived colors, taking into account the standard methods of colorimetry [2]. Numerical studies have shown that colorimetric methods are useful for better understanding of the spatial distribution of micron and sub-micron structures in butterfly wings [7].

## Experimental

**Figure 1:**
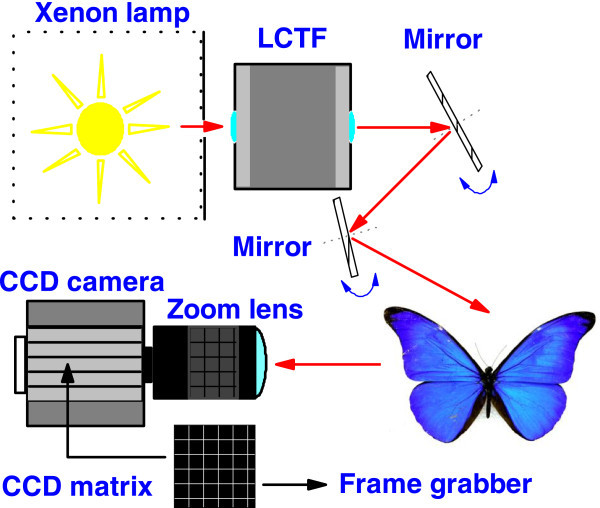
**Schematic view of the hyperspectral imaging acquisition system**. Light from the Xenon lamp converges into a LCTF and is projected into the sample using two mirrors. The mirrors also provide the fine adjustment of the illumination angle. Light reflected from the butterfly wing is recorded by a monochrome CCD camera for further processing.

Figure 1 represents a schematic view of the hyperspectral imaging system. The experimental device consisted of an illumination source or xenon lamp filtered with an ultraviolet and an infrared filter. The light was collimated using a standard convergent lens and a circular diaphragm. A liquid crystal tunable filter (LCTF) (Varispec VS-VIS2-10HC-35-SQ, Cambridge Research and Instrumentation, Inc., Boston, MA, USA) was mounted in front of the light source.

Two mirrors projected the light over the sample and provided the fine adjustment of the illumination angle. To acquire hyperspectral images, a monochrome charge-coupled device (CCD) camera (Hamamatsu ORCA C4742-95-12ER) was mounted normal to the sample so the specular component was excluded. The CCD camera had a spatial resolution of 1344 × 1024 pixels. Special modes of pixel combination (binning) were excluded. The camera also had an electronic shutter with a timer controlled by an external signal. A conventional objective was placed in the CCD camera. The images were acquired with a frame grabber (Matrox Meteor II digital PCI frame grabber). The frame grabber also provided the external signal to control the time shutter of the CCD camera. Setup, synchronization, and control of the frame grabber, the filter and the CCD camera were done using specific software in a PC. The illumination angle was fixed at 45° from surface normal, and the detection angle was at the normal. This is standard for measuring conditions in most commercial spectrophotometers (Commission Internationale de l'Éclairage, International Commission on Illumination CIE geometry 45°/0°). The entire hyperspectral system except the illumination source was shielded with dark opaque material and maintained in a dark room.

The calibration procedure and methods have been fully tested in hyperspectral data collection from natural scenes [8]. The brightly colored side of each butterfly wing sample was mounted vertically in a panel containing a black hole of 3- or 1-cm diameter. The hole ensured a proper black background within the field of view of the scene. Hyperspectral data were calibrated using a white and a black reference image. The white reference image was obtained from a white diffuser (Edmund Optics opal diffuser 50 mm). The white diffuser minimized the effect of non-homogeneous spatial distribution of intensity in the white reference image. This issue will be analyzed later. The white diffuser was not a perfect diffuser and, therefore, its reflectance factor was calibrated against BaSO_4 _using a spectrophotometer with integrating sphere (Shimadzu UV-310-PC). The black reference image provided an estimation of the dark current noise of the CCD camera and was obtained in the dark room with the sample holder empty, with the same exposure times as in the white reference image and with the light source off. The reflectance factor [2] was therefore calculated using the standard two-point correction [4,5]. The wavelength range between 400 and 718 nm was sampled at 6-nm intervals. Each hyperspectral set consisted of 54 images. The wings of two butterfly species were examined: *Morpho didius *(Nymphalidae) and *Papilio palinurus *(Papilionidae) [1,9,10]. The colorimetric methods employed in this study are standard for the representation of structural colors and are available elsewhere [2,7].

## Results and discussion

**Figure 2:**
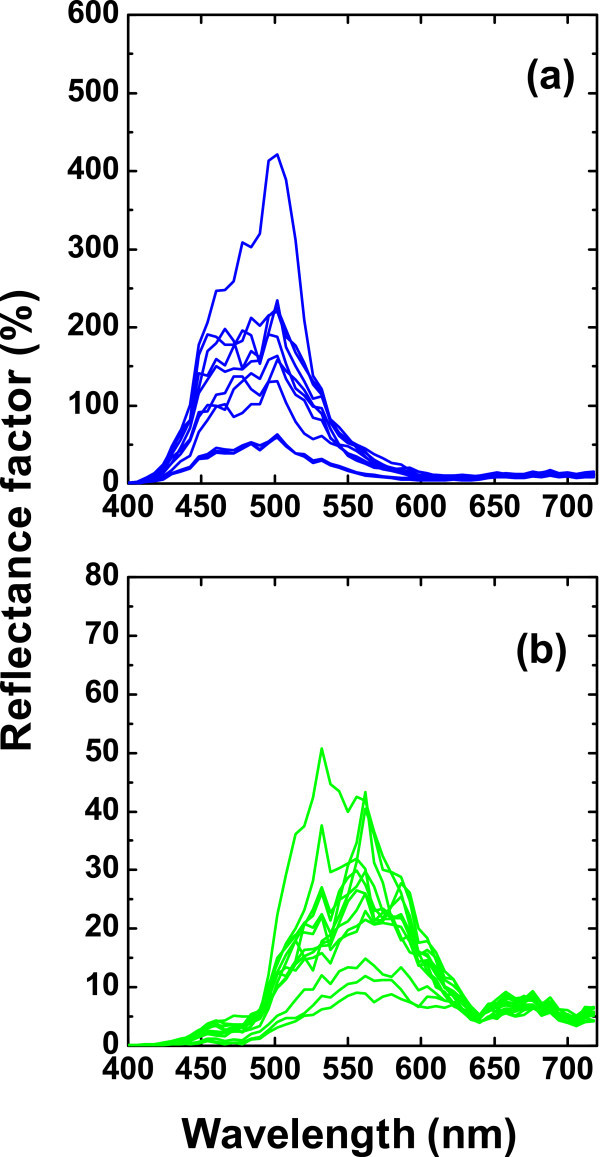
**The reflectance factor (%) as a function of the wavelength measured with the hyperspectral system. (a) **Examples of the *M. didius *(blue). **(b) **Examples of the *P. palinurus *(green). Each spectrum corresponds to the reflectance factor in a different CCD's pixel and thus in a different spatial position. In both cases, only a fraction of data is represented.

Figure 2a, b represents in a linear plot, the reflectance factor (%) for *M. didius *and *P. palinurus*, respectively. Each spectrum corresponds to the relative reflectance in each pixel and thus in a different spatial position of the butterfly wing. Only a fraction of data is displayed. The spectral profile agrees well with the data collected using conventional spectrophotometers [1,9]. Note that many reflectance values are over 100% owing to the highly directional reflectivity of the scales in comparison with the white diffuser. The spectral reflectance factor peaks at 502 nm for *M. didius *and for *P. palinurus *at 562 nm. Maximum values were 876 and 121%, respectively.

Fluctuations in the reflectance spectra come in part from the lack of spatial uniformity in the distribution of the scales in the wings as well as from the reflectance calibration. In the latter, the non-homogeneous spatial distribution of intensity in the white reference image was evaluated taking an additional white and black hyperspectral images. The reflectance factor of the white diffuser was therefore calculated following the same procedure as in the butterfly wings. It was found that variability in the reflectance spectra of the white diffuser at different pixel positions reaches a maximum reflectance factor of 23% at approximately 502 nm, then decreases, and increases again at longer wavelengths to reach a value of 21% at 718 nm. Since the reflectance factor of the *P. palinurus *was often below 10% between 620 and 718 nm, it exhibits some noise-related artifacts (see examples in Figure 2b).

**Figure 3:**
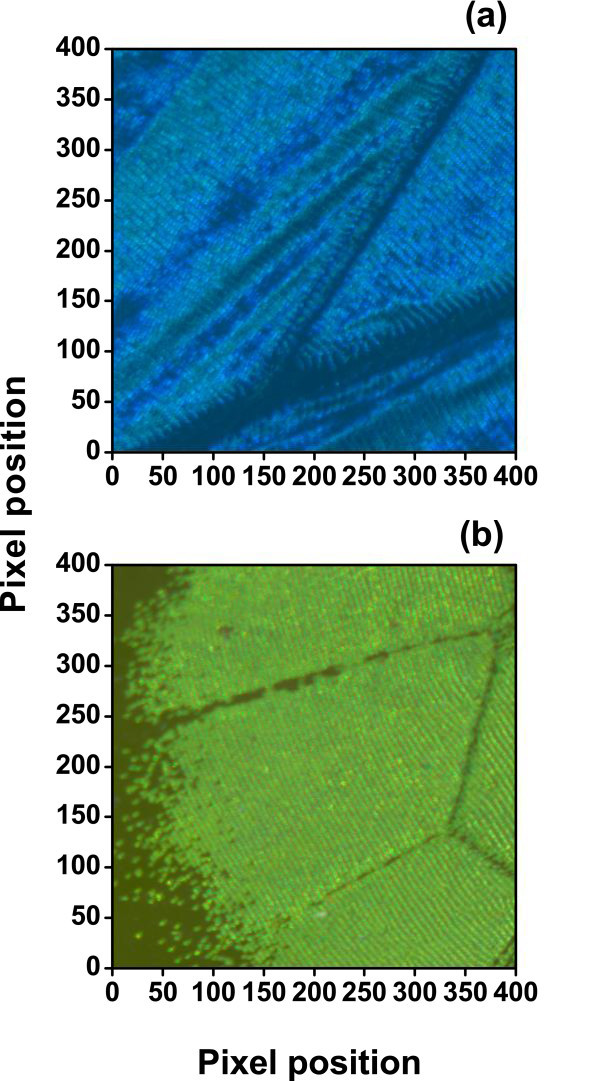
**The entire selected imaging areas in the sRGB color space (size 400 × 400 pixels)**. **(a) ***M. didius *and **(b) ***P. palinurus*. At each pixel, color coordinates were generated from reflectance spectra as the CIE XYZ tristimulus values and then converted to the sRGB color space.

**Figure 4:**
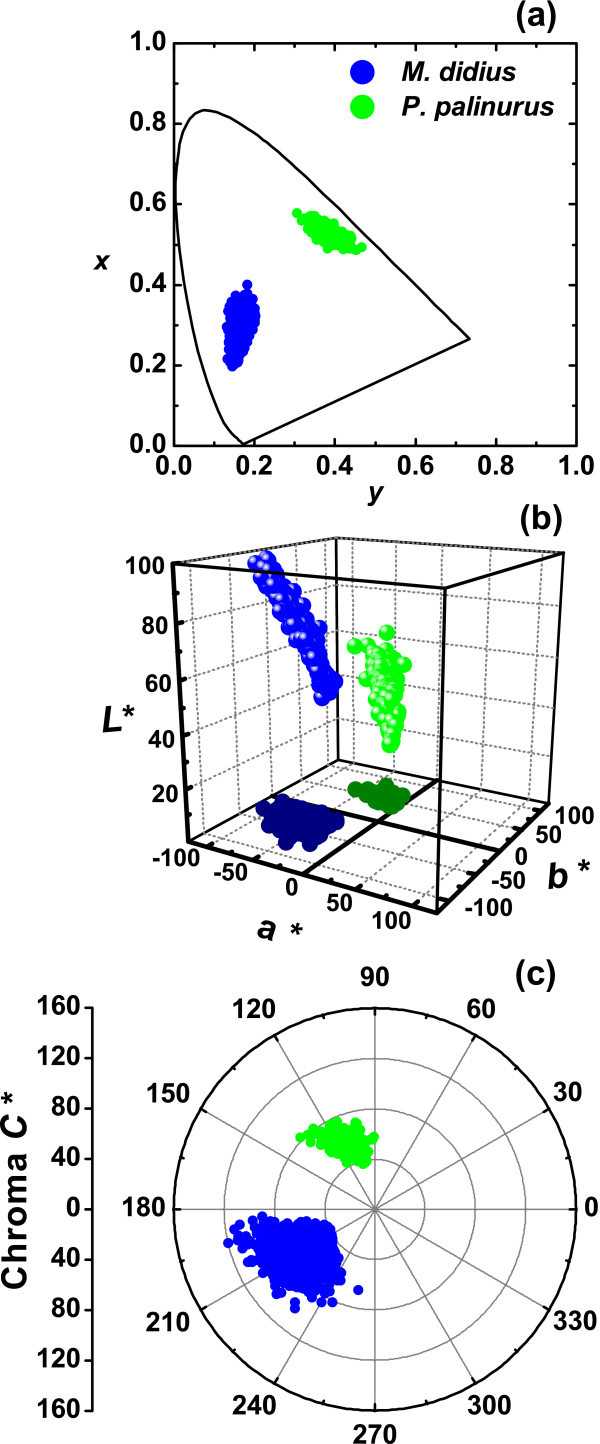
**Color coordinates generated from reflectance spectra at each pixel position. (a) **CIE-1931 chromaticity diagram. **(b) **CIELAB color space. **(c) **Polar graph of the chroma C* against the hue angle *h_ab_*. In all cases, data points show those coordinates of the *M. didius *(blue) and *P. palinurus *(green). Only a fraction of data is represented.

Figure 3a, b represents the selected area for hyperspectral imaging for *M. didius *and *P. palinurus*, respectively. The studied part of each butterfly wing covers a surface of 400 × 400 pixels at the center of the hyperspectral cube giving 1.6 × 10^5 ^reflectances. At each pixel position, the CIE XYZ tristimulus values were calculated from reflectance spectra and then converted to the sRGB color space. In both cases, the wing veins can be observed as dark-lined features. Color appearance qualitatively agrees with direct visual inspection of the samples. Figure 4a represents the chromaticity coordinates in the CIE-1931 chromaticity diagram. Figure 4b shows the three-dimensional representation in the CIELAB color space (the illuminant D65 was used). The CIELAB space is intended for the representation of pigmented coatings [2]. Alternatively, Figure 4c shows the a*b* plane in polar coordinates, with the chroma C* as the radial coordinate (related with saturation or the reciprocal of white), and the hue-angle *h_ab _*as the polar angle [2]. The results in Figure 2 indicate that variability in the reflectance spectra is mainly due to the intrinsic disorder in the structure of the butterfly wings. Figures 3 and 4 also show that irregularity is mapped in an extended color gamut. The color gamut in the *M. didius *is different from the *P. palinurus*. These color maps suggest a possible dependency with specific random arrangements of the scales and ridges such as the relative tilt angle distribution in the wing membranes [1,7]. The combination of hyperspectral imaging and accurate reflectance modeling may be important for improved understanding of the intrinsic disorder in butterfly wings and in industrial tunable structural colors.

## Abbreviations

CCD: charge-coupled device; CIE: International Commission on Illumination; LCTF: liquid crystal tunable filter; PC: personal computer.

## Competing interests

The authors declare that they have no competing interests.

## Authors' contributions

JMM performed and analyzed the experiments, drafted and revised the manuscript. SMCN and JMM prepared the hyperspectral system. SMCN revised the manuscript. PV provided the samples and revised the manuscript. All the authors read and approved the final manuscript.
